# Oral batyl alcohol supplementation rescues decreased cardiac conduction in ether phospholipid‐deficient mice

**DOI:** 10.1002/jimd.12264

**Published:** 2020-06-05

**Authors:** Hannes Todt, Fabian Dorninger, Peter J. Rothauer, Claus M. Fischer, Michael Schranz, Britta Bruegger, Christian Lüchtenborg, Janine Ebner, Karlheinz Hilber, Xaver Koenig, Fatma A. Erdem, Vaibhavkumar S. Gawali, Johannes Berger

**Affiliations:** ^1^ Center for Physiology and Pharmacology, Department of Neurophysiology and Neuropharmacology Medical University of Vienna Vienna Austria; ^2^ Department of Pathobiology of the Nervous System, Center for Brain Research Medical University of Vienna Vienna Austria; ^3^ Heidelberg University Biochemistry Center Heidelberg University Heidelberg Germany

**Keywords:** arrhythmia, batyl alcohol, cardiac impulse conduction, electrocardiography, Plasmalogens, rhizomelic chondrodysplasia punctata

## Abstract

Plasmalogens (Pls) are a class of membrane phospholipids which serve a number of essential biological functions. Deficiency of Pls is associated with common disorders such as Alzheimer's disease or ischemic heart disease. A complete lack of Pls due to genetically determined defective biosynthesis gives rise to rhizomelic chondrodysplasia punctata (RCDP), characterized by a number of severe disabling pathologic features and death in early childhood. Frequent cardiac manifestations of RCDP include septal defects, mitral valve prolapse, and patent ductus arteriosus. In a mouse model of RCDP, reduced nerve conduction velocity was partially rescued by dietary oral supplementation of the Pls precursor batyl alcohol (BA). Here, we examine the impact of Pls deficiency on cardiac impulse conduction in a similar mouse model (*Gnpat* KO). In‐vivo electrocardiographic recordings showed that the duration of the QRS complex was significantly longer in *Gnpat* KO mice than in age‐ and sex‐matched wild‐type animals, indicative of reduced cardiac conduction velocity. Oral supplementation of BA for 2 months resulted in normalization of cardiac Pls levels and of the QRS duration in *Gnpat* KO mice but not in untreated animals. BA treatment had no effect on the QRS duration in age‐matched wild‐type mice. These data suggest that Pls deficiency is associated with increased ventricular conduction time which can be rescued by oral BA supplementation.

## Graphical abstract

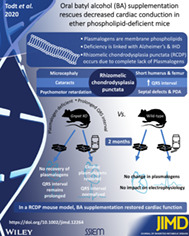

**SYNOPSIS**

In a mouse model of rhizomelic chondrodysplasia punctata deficiency of plasmalogens, a certain subtype of membrane phospholipids, produces alterations of cardiac impulse conduction which can be rescued by a dietary supplementation therapy.

## 1 INTRODUCTION

Ether lipids comprise a subgroup of phospholipids and are characterized by an ether bond at the *sn*‐1‐position of the glycerol backbone. Within the ether lipids, plasmalogens (Pls) are most abundant. Pls contain a vinyl ether bond at the *sn*‐1 position and are considered to serve a high number of important biological functions. For example, a role of Pls has been reported in modulation of lipid rafts or regulation of membrane fluidity.^1^ This could be especially important for the proper function of ion channels such as voltage‐gated sodium channels or connexins, which have been shown to localize to lipid rafts.^2^ Via this mechanism, Pls could be involved in modulating the function of the cardiovascular system and the nervous system.^3^ In the peripheral nervous system Schwann cell differentiation and myelination are regulated by Pls via proper phosphorylation of protein kinase B.^4^

The initial steps of ether lipid biosynthesis are catalyzed by enzymes localized to peroxisomes. Deficiency of ether lipids, resulting from mutations in these enzymes, gives rise to the severe congenital disorder rhizomelic chondrodysplasia punctata (RCDP). The major clinical manifestations of RCDP are microcephaly, psychomotor retardation, short humerus and femur, calcific stippling and cataract. Furthermore, a high prevalence of cardiac abnormalities has been found in RCDP patients. Thus, in a clinical study 12 of 18 patients had some form of congenital heart disease, most frequently septal defects, mitral valve prolapse and patent ductus arteriosus.^5^ Most RCDP patients die in early childhood.^6^, ^7^, ^8^, ^9^, ^10^

Several mouse models of ether lipid deficiency have been generated by targeted inactivation of one of the genes encoding biosynthetic enzymes (for review see Reference 11). The glyceronephosphate *O*‐acyltransferase knockout mouse (*Gnpat* KO), one of the models for complete ether lipid deficiency, displays phenotypic abnormalities similar to those of RCDP, including developmental defects of the central and peripheral nervous system, eyes, testis, and other organs.^12^, ^13^ Both in RCDP patients and in *Gnpat* KO mice myelination is impaired in the central and peripheral nervous system^4^, ^14^. Furthermore, these animals have reduced protein levels of connexin 43 (Cx43), a transmembrane protein involved in the formation of intercellular gap junctions.^13^ Among other functions, Cx43 is essential for the conduction of electrical impulses in the cardiac ventricles. Thus, Cx43‐deficient mice exhibit cardiac conduction abnormalities such as an increased duration of the QRS interval in the electrocardiogram.^15^, ^16^ ECG changes consistent with cardiac conduction abnormalities (first degree heart block, right bundle branch block) have also been found in RCDP patients.^5^ Interestingly, Pls are particularly enriched in the sarcolemma of cardiac myocytes.^17^ Accordingly, one aim of the current study was to investigate whether *Gnpat* KO mice exhibit prolonged electrical conduction in the heart as would be expected from the reported reduction of Cx43 levels.^13^

In another mouse model of ether lipid deficiency, the peroxin 7 (*Pex7*) KO mouse, dietary supplementation of the Pls precursor 1‐*O*‐*rac*‐octadecylglycerol (batyl alcohol, BA) rescued both biochemical and some phenotypic abnormalities. Among other organ dysfunctions, both *Pex7* KO mice and *Gnpat* KO mice develop a peripheral neuropathy with reduced motor nerve conduction velocity.^4^, ^18^ Such peripheral neuropathy has also been observed in a subset of RCDP patients.^19^ Feeding *Pex7* KO mice a diet containing 2% BA for 2 months resulted in normalization of Pls levels in peripheral tissues and improvement of the reduction in motor nerve conduction velocity.^18^ This suggests that the peripheral neuropathy in RCDP patients is at least in part caused by reversible biochemical alterations, perhaps also linked to reduced function or expression of membrane proteins such as connexins or ion channels. Hence, as a second aim we sought to determine whether possible cardiac conduction defects in *Gnpat* KO mice could be rescued by dietary supplementation of 2% BA similar to the rescue of reduced nerve conduction velocity in the *Pex7* KO mice.^18^

## 2 METHODS

### 2.1 Experimental animals

Mice with a targeted inactivation of the *Gnpat* gene (*Gnpat* ^*tm1Just*^) have been described previously.^13^ The strain was maintained on an outbred C57BL/6 x CD1 background and experimental cohorts with *Gnpat KO* (KO) and *Gnpat* ^+/+^ (WT) littermates were obtained by mating heterozygous animals. Genotypes were determined at weaning by PCR as described previously^13^ and confirmed after sacrifice. The study cohort was restricted to male animals to minimize variation evoked by gender differences. Mice were fed standard chow with food and water ad libitum and were housed in a temperature‐ and humidity‐controlled room with 12:12 hour light‐dark cycle and a low level of acoustic background noise at the local animal facility of the Medical University of Vienna. Experiments were carried out in compliance with the 3Rs of animal welfare (replacement, reduction, and refinement). All recordings and analyses were performed by experimenters blinded to genotype and treatment condition.

### 2.2 Western blot analysis

Murine hearts were weighed and homogenized in 10 volumes of lysis buffer (50 mM Tris‐Cl, pH 7.5, 150 mM NaCl, 1 mM EDTA, 1 mM ethylene glycol tetraacetic acid [EGTA], 1% NP‐40, 1% sodium deoxycholate, 0.1% SDS, 2 mM ATP, 2 mM Na_3_VO_4_, 14 mM NaF) supplied with complete protease inhibitor (Roche) using a tissue homogenizer (Polytron PT3100 equipped with a PT‐DA 3012/2 S homogenizer generator, Kinematica). Homogenates were kept on ice for 20 minutes to allow lysis and subsequently centrifuged (16 100*g*, 20 minutes, 4°C). The supernatant was removed and stored at −20°C for further analysis.

SDS‐PAGE and western blot were performed as described previously.^20^, ^21^ Quantitative analysis was performed using the Quantity One analysis software (version 4.6.6; Bio‐Rad). Primary antibodies used were rabbit α‐connexin 43 (Abcam, cat.no. ab11370; 1:30 000) and mouse α‐β‐actin (Chemicon, cat.no. MAB1501R; 1:40 000). Secondary antibodies were goat α‐mouse‐HRP (Dako; 1:20 000) and goat α‐rabbit‐HRP (Bio‐Rad; 1:20 000).

### 2.3 Treatment strategy with batyl alcohol

Test animals underwent baseline ECG recordings at the age of 12 to 13 months. Subsequently, they were randomly assigned to either the treatment or the control group. The treatment group received a standard diet (ssniff‐Spezialdiäten GmbH) supplemented with 2% (w/w) of the alkyl‐glycerol BA (Biotain Pharma Co, Ltd). The purity of BA was confirmed by nuclear magnetic resonance spectroscopy prior to treatment experiments (Department of Chemistry, University of Natural Resources and Life Sciences Vienna). Control animals received the same chow without BA. The treatment period was 2 months followed by final electrophysiological evaluation. At baseline 15 WT and 16 *Gnpat KO* animals were examined. During the treatment period, one WT and two *Gnpat KO* mice died from causes unrelated to the treatment protocol.

### 2.4 Sample preparation for lipid analysis

For homogenization of cardiac tissue, mice were sacrificed by CO_2_ inhalation and their hearts removed and perfused with a saline solution. They were weighed and homogenized in 19 volumes 155 mM ammonium bicarbonate dH_2_O using a tissue disperser (Polytron PT3100 equipped with a PT‐DA 3012/2 S homogenizer generator, Kinematica; 10 seconds, 15 000 rpm, 4°C). The samples were centrifuged at 500*g* (4°C, 10 minutes) and the supernatants stored at −80°C until further use.

### 2.5 Lipid analysis

Tissue lipids were analyzed by nano‐electrospray ionization tandem mass spectrometry (nano‐ESI‐MS/MS) using a QTRAP550 (Sciex) as described in detail previously.^23^, ^24^ All lipid data are presented as mol% of measured phospholipids.

### 2.6 ECG recordings

Mice were anesthetized with ketamine (100 mg/kg)/xylazine (10 mg/kg; single injection, i.p.), and depth of anesthesia was monitored (minimal response to hind‐foot pinch).^25^, ^26^

Murine ECGs were recorded and analyzed as reported previously.^27^, ^28^, ^29^ Small‐needle ECG leads were placed subcutaneously on all four extremities. Body temperature was maintained at 37°C using a heating pad and lamp. Signals were amplified (Gould model 11 G412301; Gould Inc., Cleveland, Ohio), high‐ and low‐pass filtered with 3 dB cut‐off frequencies of 0.3 and 1 kHz, respectively, digitized at 5 kHz, and stored for offline analysis. To reduce noise levels, ECG signals were averaged over 100 beats prior to evaluation. The duration of the QRS interval was measured from the sharp onset to the latest sharp peak of depolarization (Figure 2). In order to increase sensitivity for detecting areas of slow conduction with each experiment, only the longest QRS interval measured in any of the six standard limb leads was considered for evaluation. This method of evaluation takes into consideration that changes in conduction velocity in may have occurred both longitudinally and transversally to the myocardial fiber orientation. This may have resulted in a change not only of the speed of impulse conduction, but also of the direction of cardiac excitation. The strategy of comparing the respective longest QRS duration in any lead has been used in previous publications both in clinical and experimental studies.^30^, ^31^, ^32^ The measurements were performed by four observers blinded to the genotype of the test animals.

### 2.7 Statistics

If not stated otherwise, data are expressed as means ± SEM. Statistical comparisons were made using the two‐sample Student's *t*‐test for independent or paired samples, where appropriate.. For the statistical analysis of phospholipid levels (Figure 4), one‐way analysis of variance (ANOVA) with Dunnett's post hoc test using the “WT control” group as reference was performed. *P* values derived from post‐hoc testing after ANOVA were adjusted for multiple testing in case of control lipid classes (CE, Chol, DAG, PC, PG, PI, PS, SM, and TAG). A *P* value <.05 was considered as statistically significant.

## 3 RESULTS

### 3.1 Reduced cardiac expression of Cx43 in *Gnpat* KO mice

**FIGURE 1:**
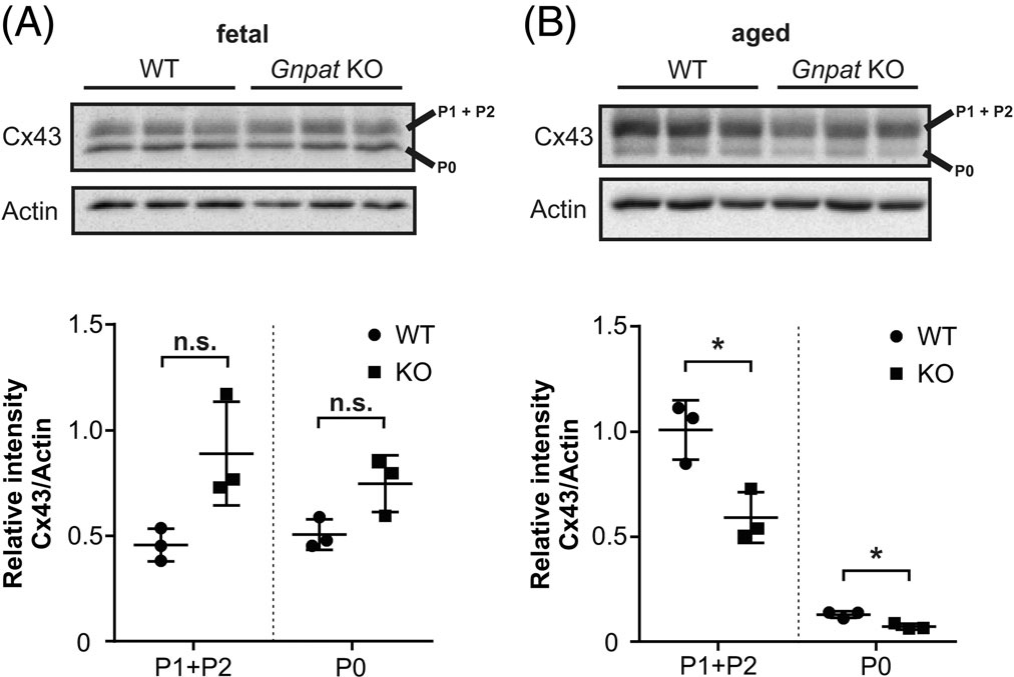
Cx43 levels in ether lipid‐deficient cardiac tissue. Western blot analysis of cardiac homogenates derived from WT and *Gnpat* KO mouse fetuses (A, *n* = 3/genotype) or aged mice (B, *n* = 3/genotype) testing for the amounts of Cx43. Immunoblots were stripped and reprobed with actin as loading control. Note the different phosphorylation variants of Cx43 (P0, P1 + P2). Due to incomplete separation between the two variants, P1 and P2 were quantified jointly. Densitometric quantification is shown as individual data together with group means ± SD. Statistical analysis was performed using two‐tailed Student's *t*‐tests followed by Bonferroni‐Holm correction for multiple comparisons. *P < .05; n.s., not significant

As mentioned above, this investigation of cardiac conduction properties in *Gnpat* KO was stimulated by the finding of reduced expression of Cx43 in this animal model.^13^ However, in that study only embryonic fibroblasts were examined for protein expression. Therefore, we sought to determine whether cardiac Cx43 expression is also affected in by Pls deficiency. As shown in Figure 1, Cx43 was unaltered between the genotypes in cardiac homogenates from fetal (embryonic day 18.5) mice (Figure 1A). However, when investigating tissue from aged mice (14‐16.5 months), we detected a striking reduction of Cx43 protein amounts by about 40% in *Gnpat* KO tissue (Figure 1B). Owing to the phosphorylation of several serine residues, Cx43 occurs in different phosphorylation states (P0, P1, and P2).^33^ The less phosphorylated P0 variant is presumed to indicate newly synthesized protein, whereas the more phosphorylated isoforms likely represent more mature variants that are either being transported to the cell surface (P1) or expressed in gap junction plaques (P2).^34^ We found that in *Gnpat* KO mice all phospho‐variants were affected to a similar extent, while the sum of P1 and P2 accounted for the major fraction of the total Cx43 protein level (Figure 1).

### 3.2 ECG parameters before treatment

**FIGURE 2:**
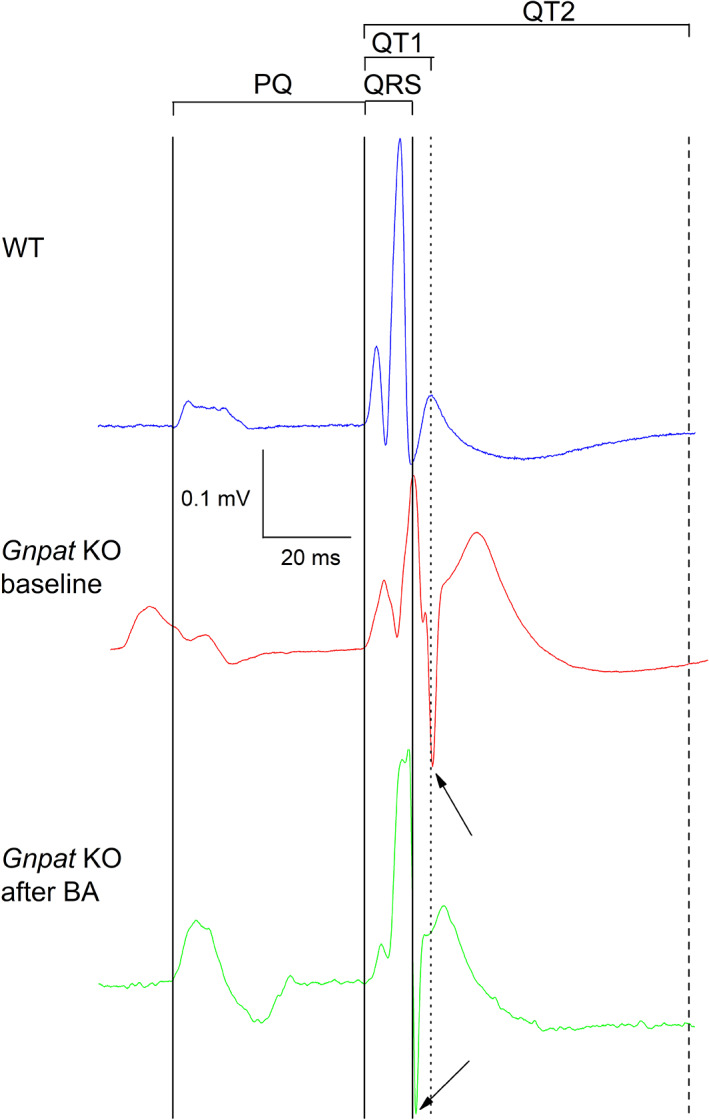
Original ECG recording from a WT (blue; upper trace) and a Gnpat KO mouse before treatment with BA (red, middle trace) and after treatment with BA (green, lower trace). The traces (lead I) are aligned with respect to the start of the QRS complex. With reference to the trace in the upper panel (blue), the solid vertical lines indicate, from left to right, the beginning of the P‐wave and the beginning and the end of the QRS complex. Furthermore, the peak of the first T‐wave (dotted line) and the end of the second T‐ wave (right) in the upper trace (WT) are indicated. The arrows indicate the end of the QRS complex in the middle trace (*Gnpat* KO at baseline, red) and the lower trace (*Gnpat* KO after BA, green). Clearly, the duration of the QRS interval and the QT1 interval are prolonged in the *Gnpat* KO mouse. However, treatment with BA in this mouse results in normalization of the QRS duration

**FIGURE 3:**
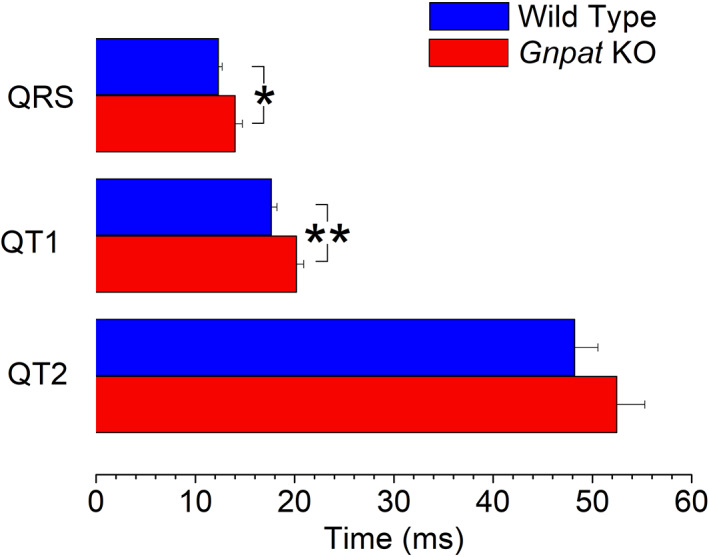
Comparison of ECG intervals in WT and *Gnpat* KO mice. Shown are the mean values from 15 WT and 16 *Gnpat* KO animals. The durations of the QRS and the QT1 intervals are significantly prolonged in *Gnpat* KO mice (*P < .05; ** *P* < .01)

To investigate, whether there are any baseline differences in ventricular conduction between the genotypes, we performed resting ECG measurements in untreated, wild‐type (WT), and *Gnpat* KO mice. The murine ECG contains at least two T‐waves representing cardiac repolarization. The first T‐wave occurs immediately after the S‐wave (“early repolarization”) and has been referred to as “J‐wave” or “b‐wave”.^35^, ^36^ A second slow wave then follows (“late repolarization”) which has been termed “c‐wave”.^36^ Both waves harbor information of cardiac repolarization.^35^ Unfortunately, in the literature both waves have often been referred to as “T‐wave”. In this article, we refer to the interval reflecting early and late repolarization as QT1 and QT2, respectively. QT1 was measured from the beginning of the QRS complex to the peak of the first slow wave following the QRS complex (Figure 2). QT2 was measured from the beginning of the QRS complex to the end of the last T‐wave. Figure 2 presents original ECG traces from a WT animal and a *Gnpat* KO mouse. With respect to WT, both QRS and QT1 intervals are prolonged in the *Gnpat* KO mouse. As shown in Figure 3, the duration of QRS intervals and QT1 intervals were significantly increased in the cohort of KO animals (13.7% and 14.4%, respectively). Furthermore, QT2 tended to increase in the KO animals, however, this change was not significant (13.7%, *P* = .4). The heart rate did not differ significantly between wild‐ type and KO animals (243.3 ± 23.2 beats/min vs 194.1 ± 29 beats/min; *P* = .2).

### 3.3 Oral BA supplementation regime

After the baseline ECG measurements, both WT and *Gnpat* KO mice were assigned to two groups. One group received unmodified food pellets (“control group”) whereas the other group was fed food pellets supplemented with 2% BA (“treatment group”). After 2 months, all animals were reassessed by ECG measurements and lipidomic analysis of cardiac tissue was performed.

### 3.4 Ethanolamine Pls are fully restored after 2 months of BA treatment in cardiac tissue

**FIGURE 4:**
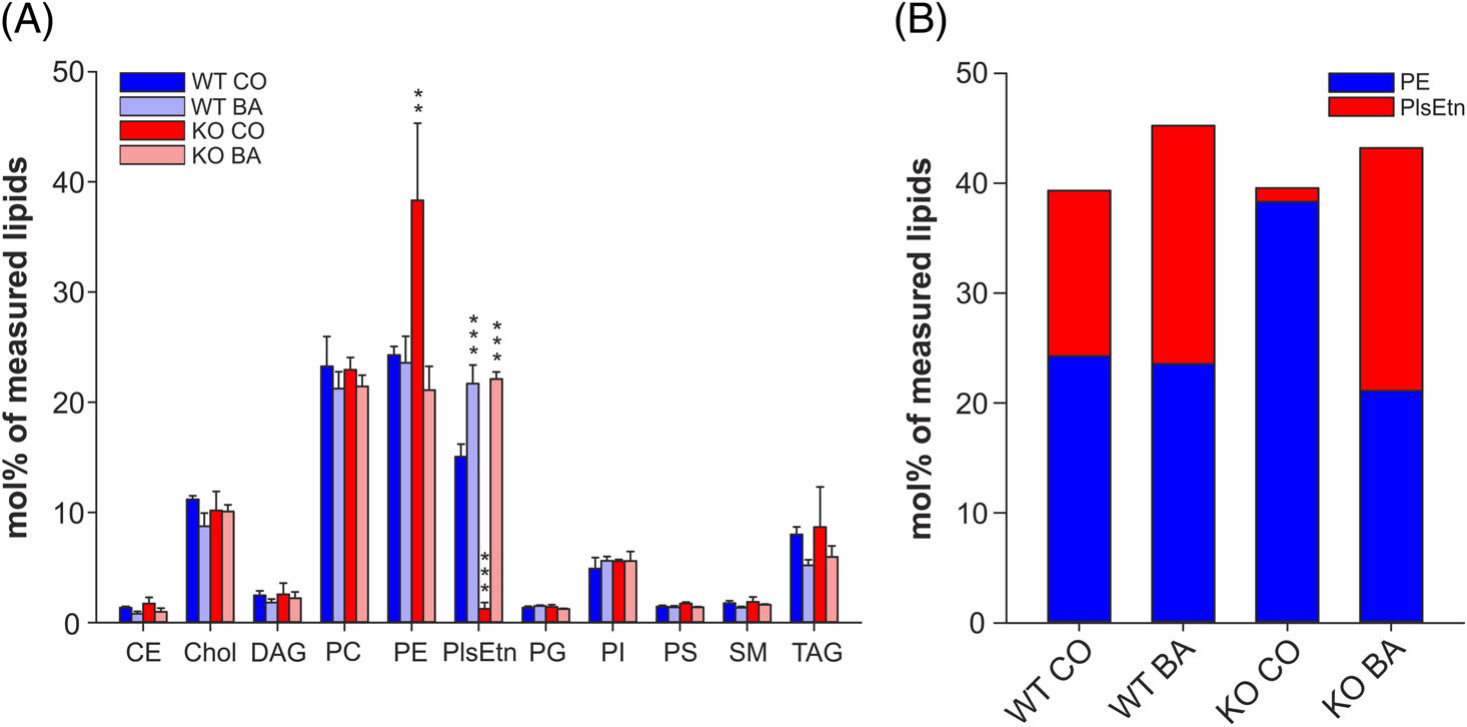
BA treatment fully replenishes ethanolamine Pls and causes compensatory changes in PE levels. A, The levels of the main membrane lipid classes were determined in cardiac tissue homogenates derived from control (CO)‐ and batyl alcohol (BA)‐treated WT and *Gnpat* KO mice by mass spectrometry. Data represent means ± SD of 3‐4 animals per group. ***P ≤ .001; **P ≤ .01 (one‐way ANOVA with Dunnett's post hoc test using WT CO as reference group; Bonferroni‐Holm correction for multiple testing in control lipid classes). B, Total ethanolamine phospholipid levels are depicted as stacked bars composed of PlsEtn and PE levels. Data consist of mean values for the two lipids as shown in A. *P =* .138 for the comparison of total ethanolamine phospholipid levels (one‐way ANOVA). CE, cholesterol ester; Chol, cholesterol; DAG, diacylglycerol; PC, phosphatidylcholine; PG, phosphatidylglycerol; PE, phosphatidylethanolamine; PI, phosphatidylinositol; PlsEtn, ethanolamine Pls; PS, phosphatidylserine; SM, sphingomyelin; TAG, triacylglycerol

To confirm the efficacy of our BA treatment regime, we analyzed the phospholipid composition in heart homogenates of treated animals by nano‐ESI‐MS/MS. As expected, the levels of ethanolamine Pls (PlsEtn), which were almost undetectable in control‐treated *Gnpat* KO hearts, were fully restored after 2 months of BA treatment (Figure 4A). In line with our previous observations in the brain of *Gnpat* KO mice and human Pls‐deficient fibroblasts,^23^ we detected a counterregulation of phosphatidylethanolamine (PE) in response to Pls levels. PE levels increased strongly in hearts of control‐treated KO animals and went back to the WT level (or slightly below) upon BA treatment (Figure 4A,B), thereby maintaining a constant level of total ethanolamine phospholipids regardless of genotype and treatment condition (Figure 4B). Remarkably, we did not notice a similar compensatory change in the level of phosphatidylcholine (PC; Figure 4A), even though Pls with a choline head group are reportedly high in cardiac tissue.^37^ However, for technical reasons, we were not able to quantify choline Pls in the current analysis. Also, we did not observe any statistically significant alterations in the levels of the other analyzed main lipid classes.

### 3.5 ECG parameters after treatment with BA

**FIGURE 5:**
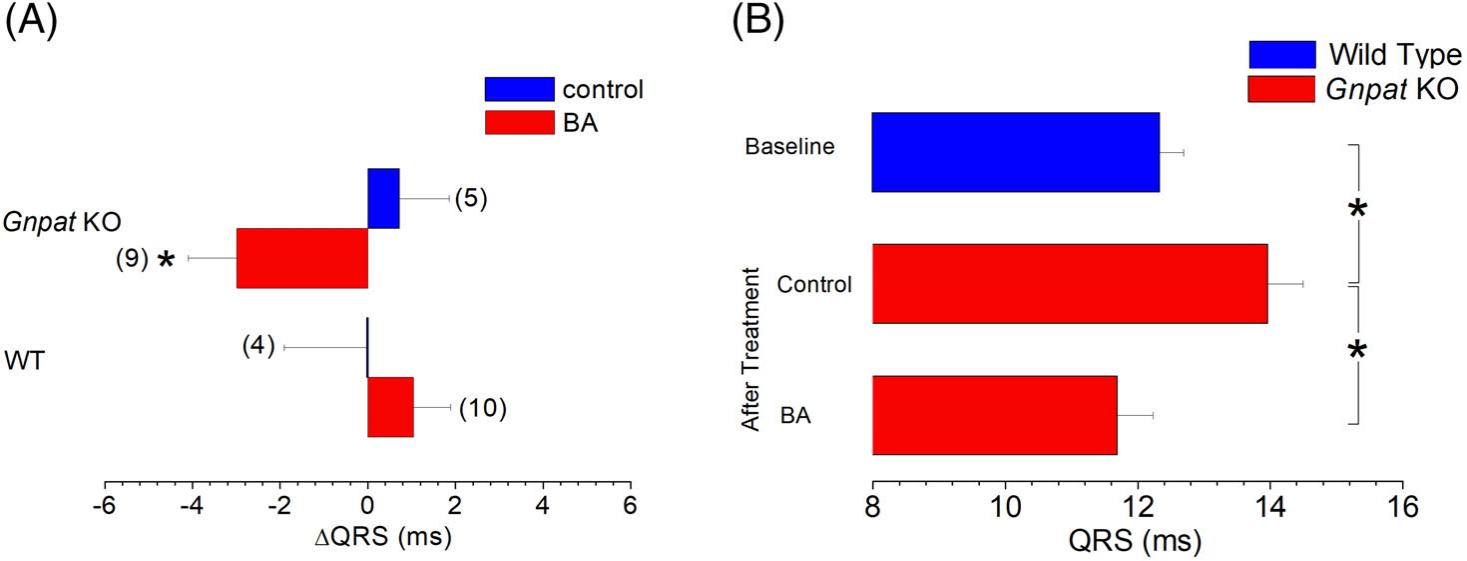
Comparison of treatment‐induced changes in the duration of the QRS complex. Bar graphs represent the difference between QRS durations before and after a 2‐month treatment with normal diet (control) or oral supplementation with BA (BA). Numbers of animals in the respective groups are indicated in brackets. When compared with the respective control group, treatment with BA induced a significant decrease in the duration of the QRS complex in *Gnpat* KO mice. Absolute values of QRS duration in WT animals before treatment (n = 15, baseline, as shown in Figure 3), and in *Gnpat* KO after treatment with unmodified food (“after treatment” control; n = 5) or with BA supplementation (“after treatment” BA, n = 9). Compared to WT animals, QRS duration remained significantly increased in *Gnpat* KO animals that were fed unmodified food. By contrast, there was no difference in QRS duration between WT animals and BA‐treated *Gnpat* KO mice

To assess possible changes in cardiac electrophysiologic properties produced by BA treatment, pairwise comparisons of QRS duration before and after treatment were performed. In both WT and KO animals, QRS duration remained unchanged with unmodified food pellets (control group) after 2 months compared with baseline. In the treatment group, QRS duration did not change in WT animals but was significantly reduced in KO animals (Figure 5A). As a result, the increase in QRS duration in *Gnpat KO* mice with respect to WT animals was abolished by treatment with BA, but not by treatment with unmodified food (Figures 2 and 5B), indicating a restoration of normal ventricular conduction in BA‐treated KO animals. All other electrophysiological parameters remained unchanged by either treatment with normal food or with BA.

## 4 DISCUSSION

Potential physiological roles of Pls include antioxidant activity and acting as substrate reservoir for the biosynthesis of eicosanoid second messengers.^38^ Apart from inherited metabolic disorders like RCDP, altered levels of Pls have been associated with a number of highly prevalent disorders such as Parkinson's or Alzheimer's disease, diabetes mellitus, arterial hypertension, ischemic heart disease, and so on (for review see References 38, 39).

This study demonstrates for the first time that, among numerous other pathologies, Pls deficiency is associated with defective cardiac conduction which can be rescued by oral BA supplementation. Previously, the only functional abnormality that responded to such dietary supplementation was a reduction in peripheral nerve conduction velocity.^18^ Intraventricular conduction delay as reflected by increases in QRS duration in the ECG is associated with increased mortality both in patients suffering from various forms of heart disease and in the general population.^40^ Presumably, slowing of intraventricular conduction may predispose to reentrant arrhythmias and to sudden cardiac death.

### 4.1 Pls deficiency is associated with reduced ventricular conduction velocity

In *Gnpat* KO mice, both QRS and QT1 intervals were significantly increased. Also QT2 intervals were also prolonged albeit nonsignificantly. Most likely, this increase in QRS interval length is a consequence of a reduction in intraventricular conduction velocity. There are several mechanisms that may account for such a decrease in conduction velocity: First, the initial depolarization of the cardiac action potential is mediated by the opening of voltage‐gated Na channels of the isoform Nav1.5. A reduction in inward current via these channels can produce QRS prolongation, for example, in conditions with reduced channel expression.^41^, ^42^ Unfortunately, no data are as yet available with regard to sodium channel expression in disease states with reduction of Pls concentration in the cell membrane. Interestingly, the activity of the cardiac sarcolemmal sodium calcium exchanger is regulated by Pls.^43^ Furthermore, the sodium calcium exchanger may interact with the cytoskeletal protein dystrophin,^44^ which, in turn, modulates the activity of voltage‐gated Na channels.^45^

Second, the conduction of the cardiac impulse is mediated via gap junctions between the myocytes.

The key gap‐junctional protein mediating the electrical connection between ventricular cardiomyocytes is Cx43. The assembly of these connexin subunits gives rise to channels that connect the intracellular compartments between adjacent cells. As mentioned previously, the protein levels of Cx43 are reduced in the *Gnpat* KO mouse fibroblasts^13^ and in cardiomyocytes from adult *Gnpat* KO mice (Figure 1B). The fact that no Cx43 reduction was observed in fetal *Gnpat* KO myocytes (Figure 1A) suggest that an aging related process is involved in the loss of Cx43 protein associated with Pls deficiency. This is not surprising because as mentioned above. A number of age‐related degenerative diseases are associated with Pls reduction. Whereas homozygous knockout of Cx43 is lethal, heterozygous knockout has been reported to increase the duration of the QRS interval.^16^ Likewise, conditional knockout of Cx43 in mice gives rise to a significant increase in the length of the QRS interval.^15^ Although Cx43 levels were not evaluated in the heart after BA supplementation, these data support the notion that reduced levels of Cx43 may account for the prolongation of the QRS interval in the *Gnpat* KO mouse. A third mechanism by which cardiac conduction may be compromised is the presence of fibrotic tissue in the myocardium.^46^ Such fibrosis may be a result of tissue necrosis and/or remodeling. Last, an increase in QRS duration may result from cardiac hypertrophy.^46^ However, in a similar cohort of *Gnpat* KO mice neither histologic evaluation of cardiac tissue samples nor in vivo magnetic resonance imaging revealed any evidence for cardiac fibrosis or hypertrophy (Dorninger et al, manuscript in preparation).

As mentioned above, *Gnpat* KO mice did not only exhibit increases in the duration of the QRS interval but QT1 also was prolonged. In man, the duration of the QT interval largely reflects the duration of cardiac repolarization, which is mainly controlled by the activity of repolarizing potassium currents. However, because of the short duration of the murine action potential, the duration of the QT interval in mice does not only reflect repolarization but also late parts of ventricular activation.^35^ Thus, the increases in QT1 may largely reflect late ventricular activation rather than prolonged repolarization.

### 4.2 Prolonged QRS duration is rescued by oral BA supplementation

In our experiments, treatment with BA resulted in complete restoration of the levels of cardiac membrane ethanolamine Pls in *Gnpat* KO animals (Figure 4). In terms of cardiac electrophysiology, QRS duration was also normalized in the treatment group (Figure 5). These results are strikingly similar to the restoration of nerve conduction velocity by the same treatment regime in *Pex7* KO mice.^18^ The molecular underpinnings of this treatment effect are unknown, but most likely the observed electrophysiological changes result from functional changes produced by normalization of the physico‐chemical properties of the cardiac cell membranes. This beneficial treatment effect also supports the notion that the pathophysiological basis of the prolonged cardiac conduction in *Gnpat* KO mice is a reduction in expression of Cx43 and/or Na channels. It is unlikely that tissue fibrosis would be responsive to such treatment (see also Reference 46).

A recent study reported decreased cardiac levels of Pls in a mouse model of dilated cardiomyopathy (DCM, induced by overexpression of Mammalian Sterile 20‐like Kinase 1). In these animals, BA supplementation for 16 weeks increased a major Pls species (p18:0) in the heart but had no effect on heart size or mechanical function assessed by echocardiography. Analysis of collagen deposition demonstrated higher levels of fibrosis in DCM hearts. Fibrosis was unchanged with BA supplementation.^47^ Unfortunately, no electrocardiographic parameters were assessed in that study. It is possible that altered electrical properties of the cardiac membrane are more likely to be restored by normalization of Pls levels than the disturbed mechanical function of the heart since the latter largely relies on the proper function of intracellular compartments and proteins such as the sarcoplasmic reticulum or the myofilaments.

### 4.3 Technical limitations of the study

This investigation was performed on animals anesthetized with a combination of i.p. ketamine and xylazine, a drug regimen that has been used previously in electrophysiologic studies with mice.^48^, ^49^ However, ketamine as well as volatile anesthetics produce block of voltage‐gated Na channels.^50^, ^51^ In theory, this Na channel blocking activity may result in slowing of cardiac conduction and prolongation of QRS duration. Therefore, we cannot completely exclude that the observed electophysiologic changes between *Gnpat* KO and WT animals may have resulted from a different response of the respective genotypes to the anesthesic agents. On the other hand, it appears unlikely that a genotype‐specific response to anesthesia may have modified the different effect of BA in the studied cohorts (Figure 5A), as the same anesthetic agents were used before and after treatment with BA or unmodified diet.

As mentioned above, a high prevalence of congenital heart disease has been found in RCDP patients.^5^ Unfortunately the presence of congenital heart disease has not been studied in *Gnpat* KO mice. Therefore, it is unknown whether the described ECG changes may have been influenced by morphological cardiac abnormalities. However, it seems very unlikely that such cardiac abnormalities may have been corrected by oral BA supplementation.

### 4.4 Previous clinical data on Pls restoration

Clinically, erythrocyte Pls levels in patients with Pls deficiency improved after BA supplementation.^52^ In several case reports beneficial effects of such supplementation has been reported on nutritional status, liver function, retinal pigmentation and motor tone.^53^ Nevertheless, large controlled studies investigating the clinical outcomes of oral supplementation therapy in this patient population are still not yet publically available. As opposed to severe congenital syndromes of ether lipid deficiency, milder forms of acquired ether lipid alterations have been reported in several diseases, for example, coronary artery disease including acute myocardial infarction.^54^ Furthermore, in these patients, prolonged QRS duration has been reported as an independent risk factor of mortality.^21^, ^55^ Perhaps restoration of normal cardiac Pls levels by oral supplementation therapy may be of clinical benefit in such diseases with acquired Pl deficiency.

## CONFLICT OF INTERESTS

Hannes Todt, Fabian Dorninger, Peter J. Rothauer, Claus M. Fischer, Michael Schranz, Britta Bruegger, Christian Lüchtenborg, Janine Ebner, Karlheinz Hilber, Xaver Koenig, Fatma A. Erdem, Vaibhavkumar S. Gawali, and Johannes Berger declare no competing interests.

## AUTHOR CONTRIBUTIONS

The experiments were conceived and designed by Hannes Todt, Fabian Dorninger, Johannes Berger, and Britta Bruegger and carried out by Hannes Todt, Fabian Dorninger, Peter J. Rothauer, Michael Schranz, Claus M. Fischer, Britta Bruegger, Christian Lüchtenborg, Vaibhavkumar S. Gawali, and Fatma A. Erdem. Data analysis was performed by Hannes Todt, Fabian Dorninger, Peter J. Rothauer, Michael Schranz, Claus M. Fischer, Britta Bruegger, Vaibhavkumar S. Gawali, Karlheinz Hilber, Xaver Koenig, and Janine Ebner. The manuscript was edited by Hannes Todt, Fabian Dorninger, and Johannes Berger.

## ETHICS STATEMENT

The present study was approved by the Institutional Animal Care and Use Committee of the Medical University of Vienna and the Austrian Federal Ministry of Science, Research and Economy (BMWFW‐66.009/0147‐WF/II/3b/2014). All institutional and national guidelines for the care and use of laboratory animals were followed.

## DATA AVAILABILITY STATEMENT

The datasets generated and/or analyzed during the current study are available from the corresponding author upon reasonable request.
